# The Functional Role of Striatal Cholinergic Interneurons in Reinforcement Learning From Computational Perspective

**DOI:** 10.3389/fncir.2019.00010

**Published:** 2019-02-21

**Authors:** Taegyo Kim, Robert A. Capps, Khaldoun C. Hamade, William H. Barnett, Dmitrii I. Todorov, Elizaveta M. Latash, Sergey N. Markin, Ilya A. Rybak, Yaroslav I. Molkov

**Affiliations:** ^1^Department of Neurobiology and Anatomy, Drexel University College of Medicine, Philadelphia, PA, United States; ^2^Neuroscience Institute, Georgia State University, Atlanta, GA, United States; ^3^Department of Mathematics and Statistics, Georgia State University, Atlanta, GA, United States

**Keywords:** striatum, reinforcement learning, striatal cholinergic interneurons, tonically active neurons, acetylcholine

## Abstract

In this study, we explore the functional role of striatal cholinergic interneurons, hereinafter referred to as tonically active neurons (TANs), via computational modeling; specifically, we investigate the mechanistic relationship between TAN activity and dopamine variations and how changes in this relationship affect reinforcement learning in the striatum. TANs pause their tonic firing activity after excitatory stimuli from thalamic and cortical neurons in response to a sensory event or reward information. During the pause striatal dopamine concentration excursions are observed. However, functional interactions between the TAN pause and striatal dopamine release are poorly understood. Here we propose a TAN activity-dopamine relationship model and demonstrate that the TAN pause is likely a time window to gate phasic dopamine release and dopamine variations reciprocally modulate the TAN pause duration. Furthermore, this model is integrated into our previously published model of reward-based motor adaptation to demonstrate how phasic dopamine release is gated by the TAN pause to deliver reward information for reinforcement learning in a timely manner. We also show how TAN-dopamine interactions are affected by striatal dopamine deficiency to produce poor performance of motor adaptation.

## Introduction

It is widely accepted that the basal ganglia play an important role in action selection, the process by which contextually appropriate actions are chosen in response to presented stimuli. To determine the appropriateness of an action, in the basal ganglia perform reinforcement learning occurs to establish action-stimulus associations. This learning process is facilitated by dopaminergic activity in the striatum, where a reward prediction error is encoded by the dopamine concentration excursion from its baseline level. When a subject performs context-appropriate actions, there is a phasic increase in striatal dopamine if the received reward is above the expectation, which means a positive reward prediction error is computed. Over time, the synapses that correspond to appropriate stimulus-action association in the striatal network are strengthened by long-term potentiation, and inappropriate actions are suppressed by long-term depression (Frank, 2005; Graybiel, 2008). Although this process is well understood from a behavioral perspective, there are still open questions about the underlying neural circuitry.

The neural populations within the striatum consist of GABAergic medium spiny neurons (MSNs), cholinergic interneurons, and GABAergic interneurons (Kita, 1993; Koós and Tepper, 1999; Tepper et al., 2010; Dautan et al., 2014; Yager et al., 2015). Many previous computational studies have focused on MSNs, which comprise a vast majority of the striatum and are heavily implicated in basal ganglia reinforcement learning (Smith et al., 1998; Kreitzer and Malenka, 2008; Wall et al., 2013). In contrast, cholinergic interneurons—also known as tonically active neurons (TANs)—comprise a small fraction of the striatal neurons and their functional role is not well understood. In this study, we integrate the results of previous studies into a computational model that includes TANs and highlight their role in propagating reward information during reinforcement learning.

Tonically active neurons (TANs) are so-called because they exhibit tonic firing activity (5~10 Hz) (Tan and Bullock, 2008; Schulz and Reynolds, 2013). TANs receive glutamatergic inputs from the cortex and thalamus (Ding et al., 2010; Yager et al., 2015; Kosillo et al., 2016). These excitatory inputs convey sensory information during a salient event or the presentation of a reward (Cragg, 2006; Schultz, 2016). When a salient event occurs, TANs generate a short burst of action potentials, which is followed by a pause in TAN activity for several hundred milliseconds. After this pause, TANs undergo a postinhibitory rebound before returning to normal levels of activity (Aosaki et al., 1994; Morris et al., 2004; Joshua et al., 2008; Apicella et al., 2011; Schulz and Reynolds, 2013; Doig et al., 2014).

TANs project to various neighboring striatal neurons and affect them by releasing acetylcholine which binds to muscarinic and nicotinic cholinergic receptors present on postsynaptic neurons. Muscarinic receptors are widely expressed in the striatal medium spiny neurons (Galarraga et al., 1999; Franklin and Frank, 2015). The nicotinic receptors are present in striatal GABAergic interneurons and axon terminals of the dopaminergic substantia nigra pars compacta (SNc) neurons (Cragg, 2006; Franklin and Frank, 2015; Shin et al., 2017; Zhang et al., 2018).

The characteristic pause in TAN activity was previously suggested to be important for conveying reward information during reinforcement learning. The TAN pause duration depends on a change in striatal dopamine concentration, which is induced by dopaminergic inputs from SNc (Maurice et al., 2004; Straub et al., 2014). This dependence exists because TANs express type 2 dopamine receptors (D2) that have an inhibitory effect on TAN activity when activated (Deng et al., 2007; Ding et al., 2010).

After a stimulus, TANs develop a slow after-hyperpolarization (sAHP) that is mainly controlled by apamin-sensitive calcium dependent potassium current (*I*_sAHP_). The sAHP lasts several seconds and induces a pause in tonic firing (Bennett et al., 2000; Reynolds et al., 2004; Wilson, 2005). Another current, the hyperpolarization-activated cation (h–) current (*I*_h_), is involved in quick recovery from sAHP. Deng et al. showed that partially blocking *I*_h_ resulted in a prolonged TAN pause duration, and that *I*_h_ was modulated by dopamine primarily via D2 inhibitory receptors (Deng et al., 2007). Thus, the duration of the TAN pause is modulated by *I*_h_ activation, which in turn is dependent on striatal dopamine concentration.

In this study, we revisit previous experimental results to formulate the following interpretations. During baseline tonic firing TANs release acetylcholine, which binds to nicotinic receptors on dopaminergic axon terminals. Thus, during their tonic firing regime, TANs exclusively define the baseline concentration of dopamine in the striatum, independently of the firing frequency of dopaminergic neurons (Rice and Cragg, 2004; Cragg, 2006). This baseline dopamine concentration corresponds to the expected reward in the determination of the reward prediction error. Furthermore, during the TAN pause, TANs stop releasing acetylcholine, thereby temporarily returning control of striatal dopamine release to dopaminergic neurons. This phasic shift in dopamine concentration corresponds to the received reward; the reward prediction error is represented as the phasic increase/decrease in dopamine concentration from the TAN-defined baseline (Cragg, 2006). Importantly, this suggests that the TAN pause serves as a time window, during which the phasic release of dopamine encodes the reward prediction error.

In this paper, we introduce a mathematical model of the TAN activity-dopamine relationship that incorporates the sAHP- and h-currents in a rate-based description of the striatal TAN population. In the model, the *I*_h_ is modulated by striatal dopamine through D2 receptor activation. Our model provides a mechanistic interpretation of the TAN activity-dopamine concentration relationship; we use our model to elucidate the mechanism by which striatal dopamine modulates the TAN pause duration, and how TAN activity regulates dopamine release. Previously, we implemented a model of reward-based motor adaptation for reaching movements that incorporated reinforcement learning mechanisms in the basal ganglia (Kim et al., 2017; Teka et al., 2017). With that model, we reproduced several behavioral experiments that involved basal ganglia-focused motor adaptation (Kim et al., 2017). Presently, we integrate our new model of the TAN-dopamine relationship into our previous reinforcement learning model. We use the integrated model to simulate striatal dopamine deficiency, as occurs in Parkinson's Disease. Even though TANs are known to send cholinergic projections to other striatal neurons, e.g., medium spiny neurons, the model does not account for these projections and focuses exclusively on the implications of interactions between TAN activity and dopamine release in striatum.

## Results

### Model of the TAN-Dopamine Relationship

Here we provide a short conceptual description of the model, sufficient for the qualitative understanding of the system dynamics. For equations and details please see Methods.

### Rate-Based TAN Population

In the model, we assume that TANs comprise a homogeneous neuronal population, whose activity is described by a single variable representing the normalized firing rate of the population. We also assume that ACh release and the activation of all cholinergic receptors in the model are proportional to TAN activity.

TANs receive excitatory inputs from the cortex and thalamus (Ding et al., 2010; Yager et al., 2015; Kosillo et al., 2016). These inputs are implemented in the model as a binary input that—when activated—initiates a burst, followed by a pause in TAN activity.

TAN activity is attenuated by the slow after-hyperpolarization (sAHP) current. The sAHP current is activated by TAN depolarization—represented in the model as TAN activity in excess of a specified threshold. The kinetics of this current are defined on a timescale of hundreds of milliseconds. This mechanism—intrinsic to the TAN population—is responsible for generating the pause in TAN activity, following a stimulus from the cortex/thalamus.

**Figure 1:**
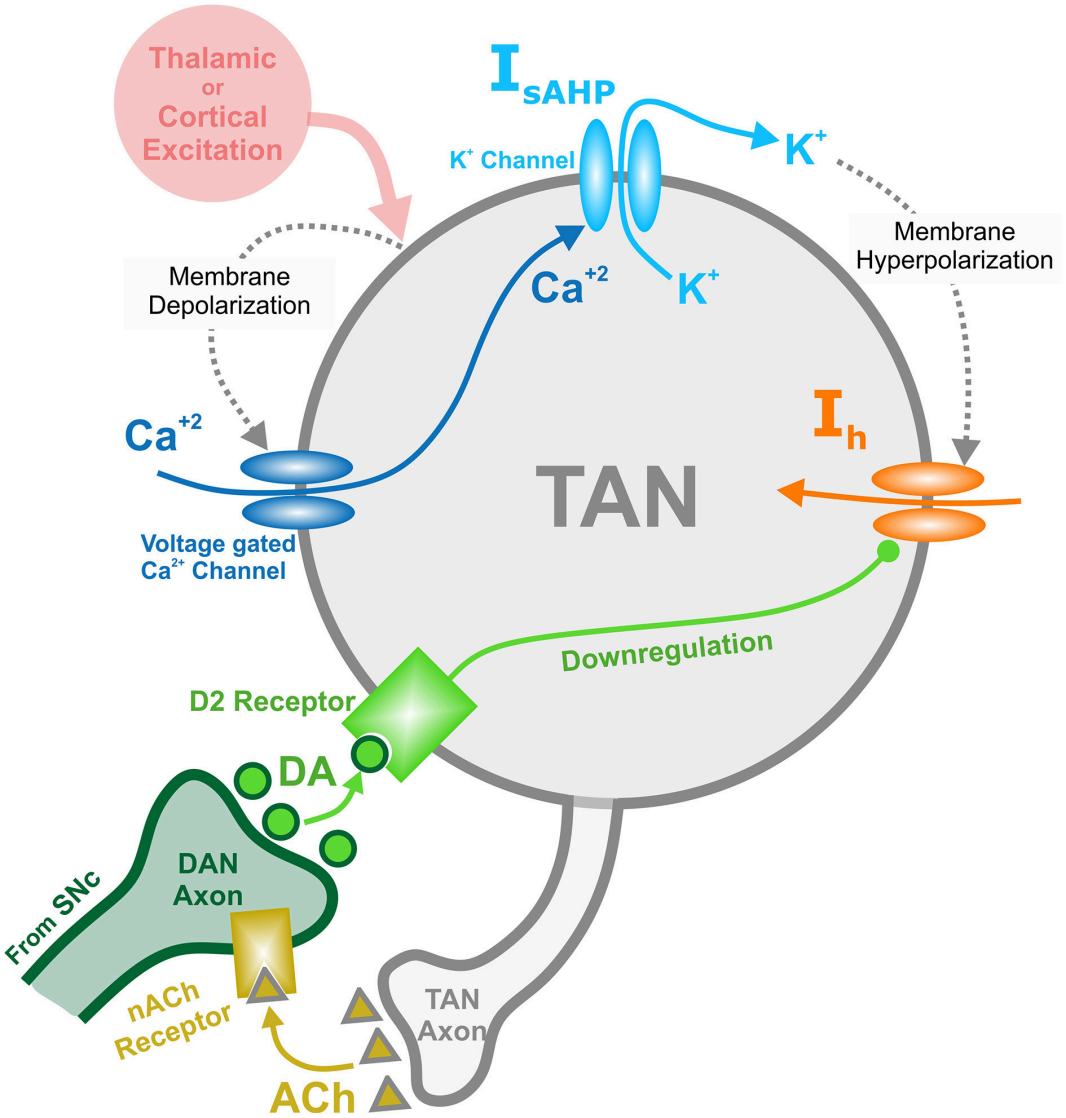
Diagram of the mechanisms involved with the TAN-dopamine release interactions. Thalamic or cortical excitation leads to membrane depolarization in TANs. In response to depolarization, calcium ions enter through voltage dependent calcium channels, and the slow after-hyperpolarization current (I_sAHP_) is activated via the efflux of potassium ions through calcium dependent potassium channels. Once the cortical/thalamic excitatory input ends, the efflux of potassium ions causes the membrane to hyperpolarize, which in turn activates the inward dopamine-dependent h-current (I_h_) that increases the membrane potential. Furthermore, dopamine (DA) from dopaminergic neurons (DANs) in substantia nigra pars compacta (SNc) binds to D2 receptors on TANs, downregulating the h-current. In concert, TANs produce acetylcholine (ACh), which binds to nicotinic acetylcholine (nACh) receptors on DAN axonal terminals. This cholinergic pathway enables TANs to modulate the release of dopamine into the synaptic cleft. Importantly—since the h-current is downregulated via activation of dopamine D2 receptors—the DA concentration affects the refractory period of TANs.

TAN activity is also affected by a depolarizing hyperpolarization-activated *h*-current. This inward current activates when TANs are hyperpolarized, and the timescale of its kinetics is similar to the sAHP current. The *h*-current thus contributes to the recovery of TANs from the pause in activity. In the model, the *h*-current deactivates in response to an increase in the concentration of dopamine—an implementation of D2-receptor agonism, which serves as a dopamine-based modulation of TAN activity (Deng et al., 2007). This mechanism provides the basis for a positive correlation between TAN pause duration and dopamine concentration. Figure 1 shows the above described mechanisms for TAN-dopamine release interaction in a diagram.

### Dynamics of Striatal Dopamine Concentration

In the model, the release of dopamine in striatum depends on the firing rate of SNc dopaminergic neurons, which receive cholinergic inputs through TAN-released acetylcholine. In the absence of acetylcholine—which occurs during a TAN pause—dopamine release is proportional to the firing rate of dopaminergic neurons. In contrast—during TAN tonic firing regimes—the release of dopamine is constant and corresponds to the baseline extracellular concentration of striatal dopamine. With increasing values of the cholinergic input to dopaminergic neurons, dopamine release becomes less dependent on the firing rate of dopaminergic neurons, and increasingly dependent on the magnitude of the TAN-provided cholinergic modulation (see Methods for mathematical description).

We also assume that the deviation of the firing rate of dopaminergic neurons from its baseline encodes the difference between the expected and received reward—the reward prediction error (Morris et al., 2004). Positive reward prediction errors correspond to increases in the firing rate of dopaminergic neurons, and negative reward prediction errors correspond to decreases in the firing rate of the dopaminergic neuron population. To constrain the model, we require that the baseline dopamine concentration is the same, whether it is defined by the baseline firing of the SNc neurons in absence of cholinergic inputs during the pause in TAN activity, or when controlled by those inputs during tonic TAN firing. We refer to deviations from the baseline dopamine concentration as “phasic dopamine release.”

**Figure 2:**
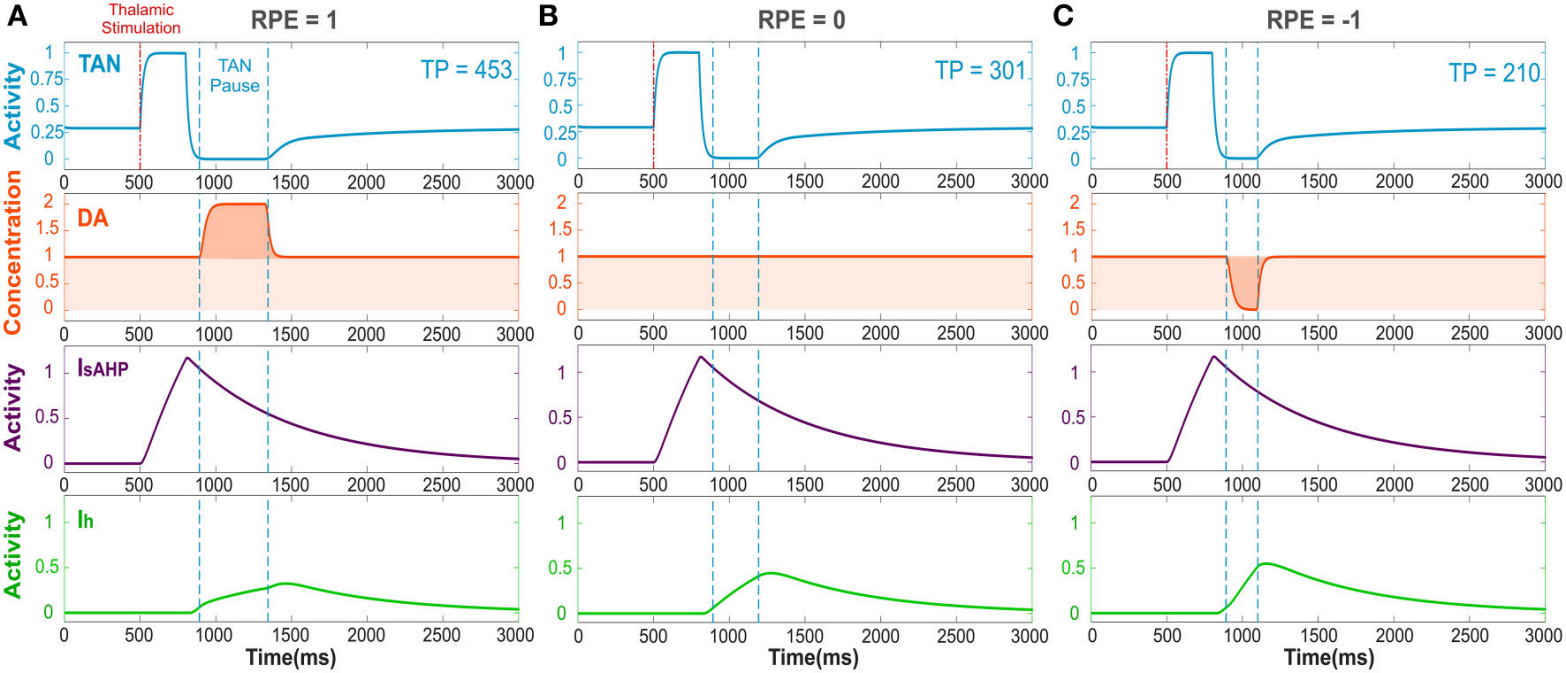
The TAN pause duration positively correlates with the reward prediction error (RPE). Thalamic stimulus induces an initial burst of TAN activity, followed by a TAN pause. The blue curve is TAN activity; the orange curve is dopamine (DA) concentration; the purple curve is the slow after-hyperpolarization current I_sAHP_ and the green curve is the h-current I_h_. **(A)** RPE = 1, the dopamine concentration increases during the TAN pause as a result of the positive RPE, which slows down I_h_ activation and thus prolong the pause. **(B)** For RPE = 0, the TAN pause is shorter, because there is no phasic change in dopamine release, so the concentration of dopamine remains at baseline during the TAN pause. **(C)** RPE = −1, the TAN pause is even shorter than for RPE = 0 because there is a net decrease in dopamine concentration during the pause, which provides the fastest I_h_ activation and hence, the shortest pause in TAN activity. Thalamic stimulation duration was 300 ms. TP stands for TAN pause duration in milliseconds.

As follows from the above, for striatal dopamine dynamics to encode the reward prediction error—i.e., for reward information to be processed in the striatum (Calabresi et al., 2000; Zhou et al., 2002; Centonze et al., 2003; Pisani et al., 2003; Cragg, 2006; Joshua et al., 2008)—a pause in TAN activity must occur. In the model (see Figure 2), a thalamic stimulus produces an initial increase in the TAN firing rate. When the stimulus ends, due to activation of the sAHP current the TAN pause begins. During the pause, TANs stop releasing acetylcholine, resulting in a phasic dopamine release—proportional to the firing rate of dopaminergic neurons. While TAN activity is paused, the sAHP current slowly deactivates, and eventually TAN activity returns to baseline (Cragg, 2006; Aosaki et al., 2010).

Figure 2 depicts the dynamics of TAN activity and dopamine concentration in cases of positive, zero and negative reward prediction error, as generated by the model. If the reward prediction error is positive, the dopamine concentration increases above the baseline during the TAN pause (Figure 2A). Since the *h*-current in TANs is inactivated via D2 agonism, the increase in dopamine release during the TAN pause prolongs the pause by suppressing the *h*-current. If the reward prediction error is zero, the dopamine concentration does not change during the TAN pause (Figure 2B), which means the pause is shorter than in the case of a positive reward prediction error. Finally, when the reward prediction error is negative, the dopamine concentration falls below the baseline during the TAN pause (Figure 2C), which upregulates the *h*-current and thus results in an even shorter pause duration. In summary, the TAN pause duration positively correlates with the reward prediction error in the model.

### Calibration of the Model

**Figure 3:**
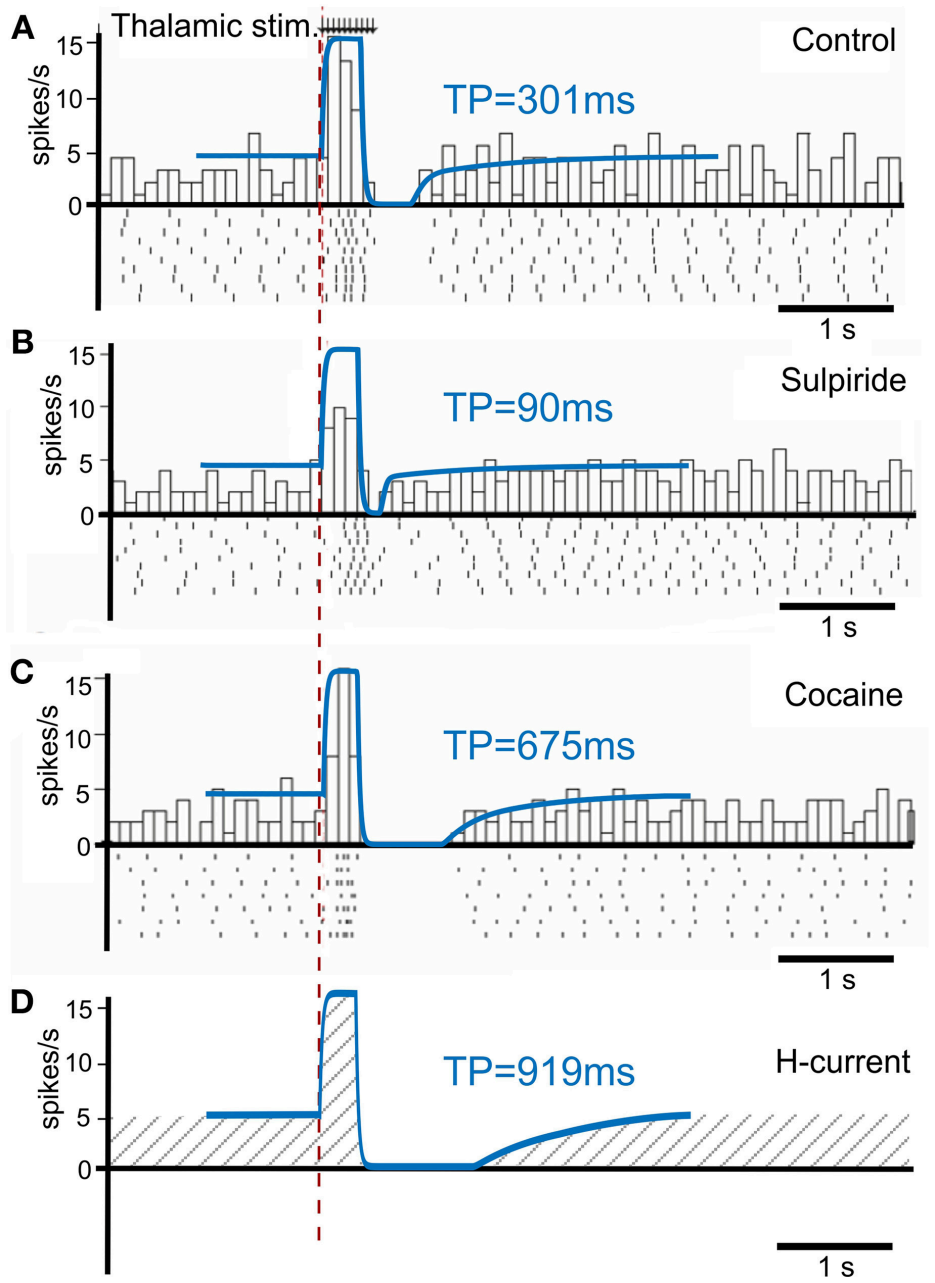
TAN activity as simulated by the model against experimental data. **(A–C)** Peristimulus time histogram (PSTH) and raster plot from striatal cholinergic interneurons in response to a train (50 Hz, ten pulses) of thalamic stimulation. The background figures were reproduced from Ding et al. (2010) with permission. For easier comparison, all simulation results (blue lines) were rescaled down at the same ratio and overlaid on the figures of experiment results. **(A)** Simulation (blue) and data (gray bars) for control condition. **(B)** Simulation and data for sulpiride (D2 receptor blockade) condition. **(C)** Simulation and data for cocaine (dopamine reuptake blockade) condition. **(D)** Simulation of the hypothetical blockade of *h*-current. TP stands for TAN pause duration.

To calibrate the model, we first simulated the condition without phasic dopamine release and compared the results to those obtained by Ding et al. (2010). They experimentally studied changes in TAN activity, which were modulated pharmacologically with drugs affecting dopamine release, reuptake, and binding (Figure 3). We varied the model parameters to reproduce the experimental time course of TAN activity in control conditions as well as after application of sulpiride and cocaine (blue traces in Figure 3).

Sulpiride is a selective D2 receptor antagonist; thus, in the model administration of sulpiride corresponds to maximal activation of *h*-current in TANs (see section Methods), which in turn shortens the pause duration. Then—because cocaine is a dopamine transporter antagonist, which results in an increase in extracellular dopamine—we simulated the cocaine condition by increasing the tonic dopamine concentration in the model until the TAN pause duration matched the experimental results.

Additionally, we performed simulations of complete suppression of *h*-current (see Figure 3D) by setting the conductance of *h*-current to zero. This simulation qualitatively corresponds to the experimental results concerned with *h*-current blockade as described by Deng et al. (2007).

### Striatal Dopamine Deficiency

Having calibrated the model, we further investigated the implications of the proposed TAN-dopamine interactions. We first simulated the condition of striatal dopamine deficiency, which may be caused, for example, by the degeneration of dopaminergic neurons in the Substantia Nigra pars compacta that occurs in Parkinson's Disease. Because dopaminergic signaling is critical for action selection and learning in the basal ganglia, dopamine deficiency adversely affects those functions.

**Figure 4:**
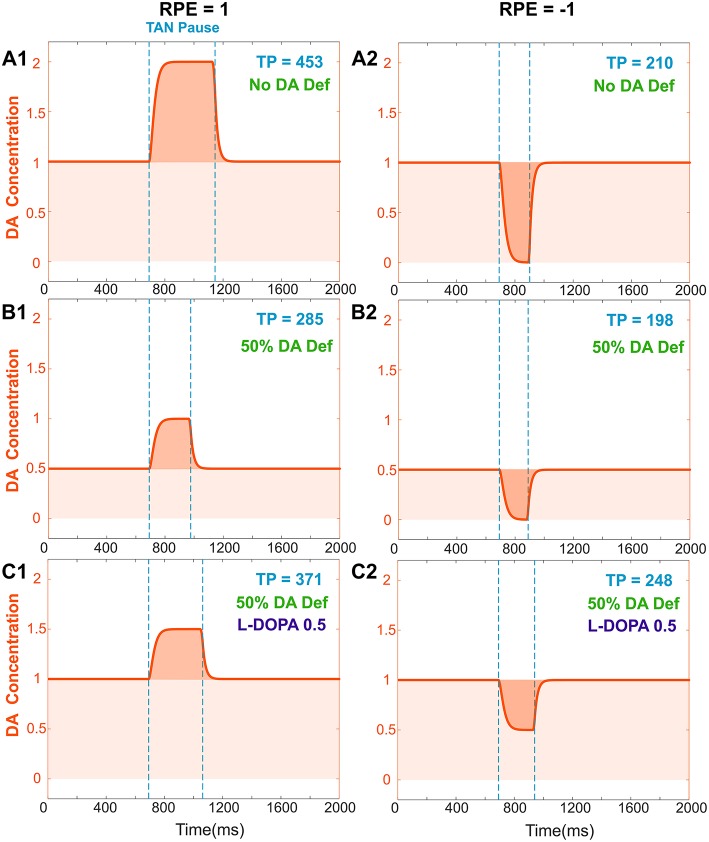
Effects of dopamine deficiency on TAN pause duration (TP, area between two dotted blue lines) and changes in dopamine concentration (orange) with/without levodopa (L-DOPA). In these simulations, a 50% dopamine deficiency (DA Def) causes both the baseline dopamine concentration and the phasic dopamine release to decrease. **(A1–2)** RPE = 1 and −1, no dopamine deficiency for reference. **(B1)** RPE = 1, 50% dopamine deficiency. Normally, the baseline concentration of dopamine would be 1.0. With a deficiency of 50% of dopaminergic inputs, the baseline dopamine concentration is exactly halved; additionally, the phasic release of dopamine decreases in magnitude by 50%, and therefore the duration of the TAN pause also decreases. **(B2)** RPE = −1. The tonic and phasic release of dopamine are both reduced by the 50% due to dopamine deficiency. During the pause, dopamine concentration converges to zero, so the pause is similar (slightly shorter) to **(A2)**. **(C1)** RPE = 1. When levodopa (0.5) is applied, the baseline concentration of dopamine returns to normal (1.0) and the duration of the TAN pause duration increases, but it remains smaller than the one with no DA deficiency **(A1)**. This is because the magnitude of phasic dopamine release is unaffected by levodopa. **(C2)** RPE = −1. When levodopa (0.5) is applied, the baseline concentration of dopamine returns to normal (1.0) as for RPE = 1, but the duration of the TAN pause exceeds the one with no DA deficiency **(A2)**. This is due to the increased (non-zero) dopamine concentration during the pause.

We assumed that the degenerated Substantia Nigra pars compacta neuronal population releases less dopamine during both tonic and phasic modes. Accordingly, dopamine deficiency conditions were simulated by reducing the tonic dopamine concentration by a factor <1 and reducing the reward prediction error by the same factor (see section Materials and Methods). Thus, both tonic (baseline) and phasic dopamine levels are decreased by the same factor; Figures 4A,B show changes in TAN pause and dopamine dynamics in dopamine deficiency conditions. Noteworthy, in the dopamine deficiency conditions, the duration of the TAN pause decreases in response to the reduction in dopamine concentration (Figure 4).

### Effects of Levodopa Medication

Using the model, we investigated the mechanisms of levodopa-based treatments for dopamine deficiency. Levodopa (L-DOPA) is a common medication for Parkinson's Disease patients to increase overall dopamine concentration in the brain (Brooks, 2008; Kalia and Lang, 2015). Levodopa readily passes across the blood brain barrier and converted to dopamine (Wade and Katzman, 1975; Hyland and Clayton, 1992). This additional extracellular dopamine propagates nonspecifically throughout the brain. When simulating levodopa treatment conditions, we assume that levodopa administration increases the tonic (baseline) dopamine concentration but does not affect the phasic dopamine release.

In the model, the concentration of levodopa is represented as a constant added to the baseline dopamine concentration. Figure 4C shows the corresponding simulation results. Importantly, although phasic dopamine release is unaffected by levodopa, the increase in tonic dopamine prolongs the TAN pause duration.

### Non-error-based Motor Adaptation During Dopamine Deficiency

In addition to our analysis of the local effects of dopamine deficiency on the striatal dopamine concentration, we also simulated the effects of dopamine deficiency on motor adaptation by incorporating the current model of TAN-dopamine interactions into our previously published model of reward-based motor adaptation (Kim et al., 2017) (see section Materials and Methods for details). Using this integrated BG model—including the TAN-dopamine interactions—we reproduced the non-error based motor adaptation experiments of Gutierrez-Garralda et al. (2013).

**Figure 5:**
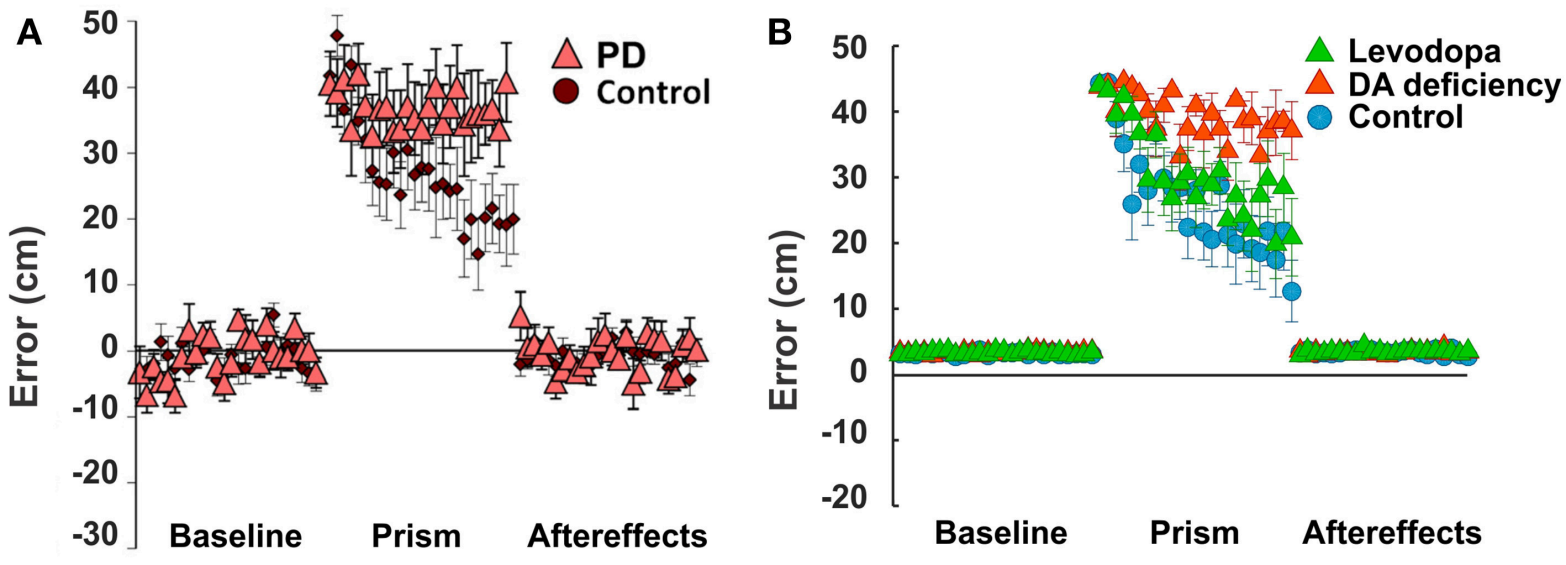
Non-Error based motor adaptation in 50% of dopamine (DA) deficiency condition with/without levodopa medication. **(A)** Results of ball throwing tasks performed by healthy people and Parkinson's Disease (PD) patients. During experiment, a dove prism was used to horizontally flip subjects' vision as perturbation. This figure was adapted from Gutierrez-Garralda et al. (2013) with permission. **(B)** Simulation results with levodopa medication. Levodopa means the condition of 50% dopamine deficiency with levodopa medication ([LDOPA] = 1.0). Colored center markers (triangle or circle) are average error values of 8 sessions and error bars represent standard errors. 1 session = 75 trials (Baseline = 25 trials, Prism (visual perturbation) = 25 trials and Aftereffects = 25 trials).

In these experiments, healthy subjects, Parkinson's Disease patients, and Huntington's Disease patients threw a ball at a target under different visual perturbation scenarios. In one scenario, each subject's vision was horizontally reversed using a Dove prism so that missing the target to the right was percived as missing to the left, and vice versa—corresponding to a sign change in the percieved error vs. the actual error. This perturbation rendered error-based motor adaptation useless. In these experiments, each session was comprized of 75 trials (25 trials before the perturbation, 25 trials with the pertubation, and 25 trials after the perturbation). Eight sessions per subject were performed and averaged. Subjects in the control group gradually overcame the visual perturbation and reduced the distance error, but Parkinson's Disease subjects showed poor learning performance (distance errors fluctuated without any sign of adaptation in 25 trials, Figure 5A).

In our simulations, we assumed that dopamine deficiency was the cause of Parkinson's Disease symptoms (Kalia and Lang, 2015). To see how much dopamine deficiency affects learning performance in the model, we performed multiple simulations with changing dopamine deficiency conditions from 0 to 90% (see section Methods for Details). The simulation of 0% dopamine deficiency (Figure 5B, control) shows a trend of decreasing errors, which accurately reproduces the experimental results of control subjects in Gutierrez-Garralda et al. (2013) (Figure 5A, control). As we can see in Figure 5B (Dopamine Deficiency), at 50% dopamine deficiency, learning performance is poor and is similar to the experimental results in Parkinson's Disease patients (Figure 5A, PD). For over 50% dopamine deficiency, average distance error remains at the initial level for all 25 trials, while error fluctuation and standard distance error decrease (result not shown). In summary, almost no learning occurs in the model when dopamine deficiency exceeds 50%.

### Recovery of Non-error-based Motor Adaptation With Levodopa

To investigate the effects of levodopa medication on reinforcement learning in the striatum, again we simulated the same experimental settings. In the model, dopamine deficiency was set at 50% to simulate Parkinson's Disease conditions and simulations were performed with varying levodopa values representing additional striatal dopamine converted from levodopa medication. Figure 5B (Levodopa) shows the simulation results.

At levodopa values corresponding to 100% recovery of the baseline dopamine concentration, the average error decreases siginificantly at the end of the perturbation trials (Figure 5B, Levodopa). Thus, the overall learning perfomance of the model significantly improves as a result of levodopa administration.

However—although the learning performance improves—the performance of levodopa-medicated patients is still noticably worse than in control subject simulations. This performance difference can be easily understood in the context of our model of TAN-dopamine interactions. In the model, when levodopa is introduced, the tonic concentration of dopamine returns to healthy baseline levels, but the amplitude of phasic dopamine release is not recovered (compare Figures 4A1,C1). Therefore, our integrated model simulations suggest that Parkinson's patients can partially regain learning performance following levodopa administration—due to the increase in tonic dopamine concentration—but a full recovery is impossible without a corresponding increase in phasic dopamine release.

## Discussion

In this study we investigated the relationship between striatal dopamine and TAN activity; specifically, we elucidated the mechanism by which this interaction affects reinforcement learning in the striatum. Striatal TANs temporarily pause their tonic firing activity during sensory or reward events. During tonic firing regimes, TAN activity defines the baseline striatal dopamine concentration via nicotinic ACh receptors (nAChR) activation on dopaminergic axon terminals (Rice and Cragg, 2004); thus, the TAN pause enables a temporary variation of dopamine release. The duration of the TAN pause is important as it creates a window of opportunity for the dopaminergic neurons to transmit information about the reward prediction error by phasically modulating the dopamine concentration in the striatum. In turn, the concentration of dopamine determines the duration of the TAN pause by modulating the *h*-current via D2 receptors in TANs (Deng et al., 2007). Accordingly, in our model, the TAN pause enables the phasic release of dopamine, and the duration of the TAN pause varies with dopamine concentration.

One of the objectives of this study was to extend our previous model by adding details of the striatal circuit concerned with cholinergic modulation of dopamine release. By doing so, we were able to investigate how TAN activity contributes to reinforcement learning mechanisms in simulated behavioral experiments.

In the model, phasic dopamine levels are defined by the activity of dopaminergic neurons, which codes the reward prediction error. Deviations of striatal dopamine concentration from its baseline underlie the plasticity of cortico-striatal projections to medium spiny neurons, representing a basis for reinforcement learning in the striatum. These deviations last for the duration of the pause in TAN activity. Therefore, the magnitude of long-term potentiation or depression of cortico-striatal projections depends on the pause duration, which may affect learning performance.

TANs express D2 dopamine receptors, which are inhibitory. Through this mechanism, the duration of the pause in TAN activity positively correlates with striatal dopamine concentration. In conditions of dopamine deficiency, the baseline dopamine concentration is reduced, which also shortens the duration of the TAN pause.

Based on our model predictions, we speculate that levodopa medication improves learning performance in Parkinson's patients by increasing the baseline dopamine concentration and thus prolonging the pause in TAN activity—even though the magnitude of phasic dopamine excursions may be not affected by this medication.

### Dopamine Release and Cholinergic Regulation

Within the Substantia Nigra pars compacta—a structure in the midbrain—are dopaminergic neurons that project to the striatum. These dopaminergic neurons are known to encode reward-related information by deviating from tonic baseline activity (Schultz, 1986; Hyland et al., 2002). Striatal dopamine release occurs via vesicles at local dopaminergic axon terminals (Sulzer et al., 2016). However, the amount of dopamine released is likely to be not always defined by the firing rate of the presynaptic neuron.

Cholinergic activity plays a major role in modulation of dopamine release in the striatum. For example, synchronized activity of striatal TANs directly evokes dopamine release at the terminals—regardless of the activity of dopaminergic neurons (Cachope et al., 2012; Threlfell et al., 2012). TANs release acetylcholine (ACh), which binds to nicotinic receptors on the axons of dopaminergic neurons—and when these cholinergic inputs are activated, dopamine release is independent of electrical stimulation frequency (Rice and Cragg, 2004). However, when these nicotinic receptors (nAChRs) are blocked, the magnitude of dopamine release becomes proportional to the stimulation frequency (Rice and Cragg, 2004). Therefore, it is necessary for the cholinergic inputs to dopaminergic neurons to cease so that dopamine release reflects the firing activity of the presynaptic neurons.

Our model assimilates the above observations via the following assumptions. Baseline striatal dopamine concentration is determined by the presynaptic action of ACh on dopaminergic terminals (Threlfell et al., 2012) through nAChR desensitization. With no cholinergic inputs, e.g., when TAN activity ceases or nAChRs are blocked, the firing rates of dopaminergic neurons define the dopamine release. In other words, the phasic component of dopamine release is determined by Substantia Nigra pars compacta activity, which codes the reward prediction error. Therefore, the functional role of the pause in TAN activity is to allow the striatal dopamine concentration to vary, thus creating a window of opportunity for dopaminergic neurons to deliver the reward information to and enable reinforcement learning in the striatum.

Variations in the phasic release of dopamine reflect the reward prediction error (Hollerman and Schultz, 1998; Schultz, 1998, 1999); thus, in the case that the reward received is exactly the same as the expected reward—reward prediction error is zero—the dopamine concentration should not change during the TAN pause. In the model, as explained above, the baseline dopamine concentration is constrained by cholinergic inputs from TANs, and during the pause, dopamine release is controlled by the firing rate of dopaminergic neurons in the Substantia Nigra pars compacta. Therefore, we constrained the model by requiring that Substantia Nigra pars compacta firing corresponding to a reward prediction error value of zero (RPE = 0)—in absence of cholinergic input during the pause—leads to exactly the same dopamine release as during normal TAN activity. The exact homeostatic mechanisms responsible for such tuning remain open for speculation.

In our model, we did not differentiate between different parts of striatum in terms of cholinergic regulation of dopamine release. However, it was reported that the nucleus accumbens shell, the most ventral part of striatum, has a distinctive modulation mechanism of dopamine release with much higher activity of acetylcholinesterase minimizing nAChR desensitization, which is different from nucleus accumbens core and dorsal striatum (Shin et al., 2017). There is also evidence that DA release in nucleus accumbens is modulated by ACh not only through nicotinic but also via muscarinic receptors of several types activation of which has different effects on DA concentration (Shin et al., 2015). Our model does not account for this.

In our model, we focused on the functional role of TAN activity-dopamine interactions in reinforcement learning. Thus, we did not consider the effect of TANs on other striatal neuron types. For example, MSNs are known to receive cholinergic inputs via muscarinic M1 and M2 receptors. Functional role of these projections was discussed elsewhere. In particular, other computational models proposed that TANs might have a timing control function to hold and release MSNs (Ashby and Crossley, 2011; Franklin and Frank, 2015). Besides TANs and MSNs, many other types of interneurons have been identified in striatum, such as parvalbumin fast spiking interneurons, neuropeptide Y interneuron, calretinin interneurons, Tyrosine Hydroxylase interneurons (Tepper et al., 2010, 2018; Xenias et al., 2015). Functional roles of these interneurons and their relationships with cholinergic interneurons are not clearly understood. However, this does not rule out the possibility, that some of these neuron types interact with TANs and thus may play a role in TAN activity regulation.

### TAN Pause Duration

In the model, the pause in TAN activity is initiated by transient excitatory corticothalamic inputs. Furthermore, the duration of the pause is dependent on the extracellular dopamine concentration (Deng et al., 2007; Oswald et al., 2009; Ding et al., 2010). To replicate this dependence, we calibrated the duration of TAN pause in the model to *in vitro* experimental data from Ding et al. (2010).

It is important to note that longer thalamic stimulation means stronger activation of the slow after-hyperpolarization (sAHP) current, and hence more time is required for its subsequent deactivation. This prediction is consistent with the *in vitro* studies by Oswald et al. In their experiments, a higher number of stimulation pulses did generate stronger after-hyperpolarization in TANs below their resting potential—and accordingly evoked a longer pause in TAN activity. In addition, several *in vitro* and *in vivo* experiments agree that the magnitude of thalamic input positively correlates with the TAN pause duration (Oswald et al., 2009; Schulz et al., 2011; Doig et al., 2014). Although we cannot directly compare our simulation results with their data, our TAN model exhibits a qualitatively similar relationship between input duration and pause duration.

**Figure 6:**
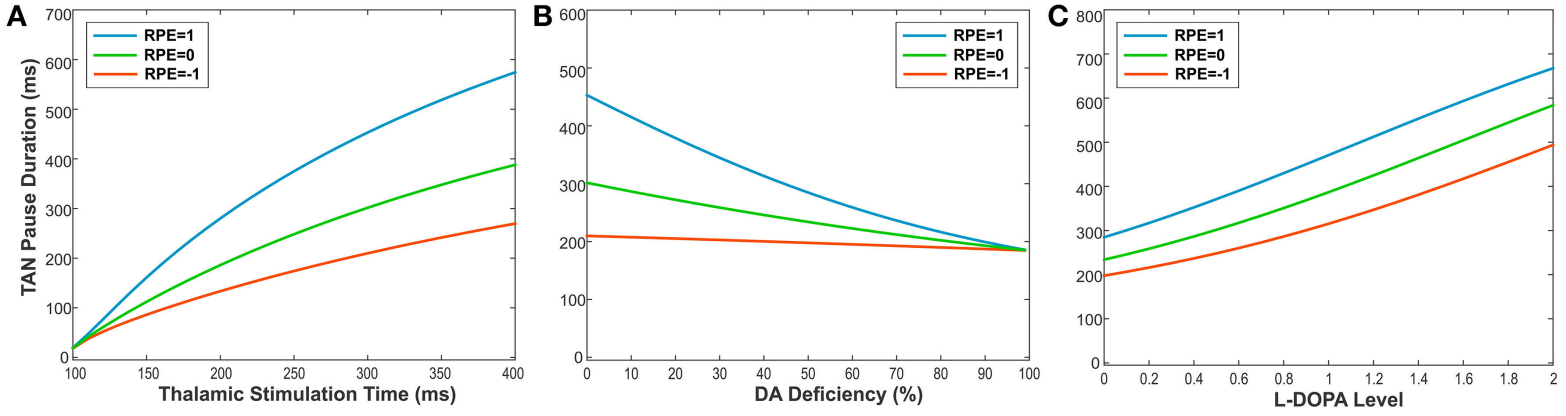
**(A-C)** The changes in TAN pause (TP) duration by three different factors: the duration of thalamic stimulation, the percentage of dopamine (DA) deficiency, the L-DOPA level in 50% DA deficiency condition when RPE (Reward Prediction Error) = 1 (phasic, reward), 0 (tonic baseline), and −1 (phasic, aversive), respectively. **(A)** The changes in TP duration by the duration times of thalamic stimulation. The increment of thalamic stimulation duration increases TP duration for all RPE values. The difference of TP duration between RPE = 1 and RPE = −1 keeps increasing nonlinearly as increases in thalamic stimulation duration. **(B)** The changes in TP duration by the percentages of DA deficiency. The increased percentage of DA deficiency decreases TP duration when RPE = 1 and 0. For RPE = −1, the TP duration is nearly independent of the amount of DA deficiency, which is the result of RPE = −1 corresponding to the minimum possible DA concentration during the TP. Therefore, the TP duration for RPE = −1 is unaffected by the degradation of dopaminergic inputs. The deviation difference of TP duration from RPE = 0 between RPE = 1 and RPE = −1 keeps decreasing nonlinearly as increases in percentage of DA deficiency, which means minimizing the time difference between reward and aversive conditions for reinforcement learning and in turn deteriorating the learning performance. **(C)** The changes in TP duration by the levels of L-DOPA in 50% DA deficiency condition. In response to the administration of L-DOPA, the TP duration increases similarly for all RPE values. This follows from the fact that L-DOPA alters the baseline concentration of dopamine, but does not affect the phasic dopamine release.

To illustrate this relationship, we performed simulations, varying the duration of thalamic stimulation (from 100 to 400 ms) as shown in Figure 6A. The duration of the TAN pause increases non-linearly in response to increasing thalamic stimulation duration. Interestingly, this increase in the pause duration is stronger for higher reward prediction error values, which is because of the larger phasic dopamine concentration when the reward prediction error increases. The reward prediction error is independent of the thalamic stimulus duration, and the pause duration is sensitive to both variables. Thus, we manipulated each variable independently to show the dependence of the pause duration on both.

Furthermore, the TAN pause duration is dependent on any change in the extracellular dopamine concentration—not just the RPE-determined phasic dopamine release. Therefore, we also produced simulations demonstrating the effects of dopamine deficiency as well as the effect of levodopa administration on the TAN pause duration. Importantly, dopamine deficiency has almost no effect on the TAN pause duration when the reward prediction error is at a minimum (see the orange line in Figure 6B). This model behavior follows from the observation that the reward prediction error correlates with the magnitude of phasic dopamine release. If the reward prediction error is at its minimum possible value (in our model, RPE = −1), then neither the amount of phasic dopamine nor the duration of the TAN pause can be decreased by dopamine deficiency conditions. In contrast, the administration of levodopa affects the TAN pause duration without any dependence on the reward prediction error. This follows from the fact that levodopa alters the baseline concentration of dopamine—not the phasic dopamine release—which is not dependent on the reward prediction error.

### Comparisons With Other Models

The model presented here is not the first computational model of TAN activity. For example, Tan and Bullock previously developed a computational model incorporated *h*-current as an intrinsic property of TANs (Tan and Bullock, 2008). Their model was also a non-spiking model that focused on the generation mechanism of TAN-specific activity patterns, which the authors attributed to intrinsic TAN properties. Even though their model accounted for modulation of TAN activity by dopamine level, it did not include a mechanism that affects the dopamine release, which our model did.

Ashby and Crossley also developed a BG model that included Hodgkin-Huxley style spiking TANs with *h*-current (Ashby and Crossley, 2011). Their model emphasized the inhibitory effect of TAN activity on striatal medium spiny neurons (MSNs) through muscarinic receptors. They proposed that tonic TAN activity normally suppresses MSN firing, which is released during the TAN pause. Similar idea was exploited in the computational model of BG circuits by Franklin and Frank (2015) who proposed that the pause in TAN activity is formed by local striatal inhibition to code the uncertainty and regulate learning rates through cholinergic projections to MSNs. The model we propose significantly differs from these two models with respect to the gating function of the pause in TAN activity. Our model focuses on cholinergic dopamine regulation and does not incorporate direct cholinergic projections to—or GABAergic projections from—MSNs.

To the best of our knowledge, the model proposed here is the first that incorporates bidirectional effects of cholinergic and dopaminergic signaling in the striatum and explores the implications of these interactions by simulating real and hypothetical behavioral experiments in realistic settings. This was made possible by embedding our implementation of TAN-dopamine interactions into the model of reward-based motor adaptation we previously published (Kim et al., 2017).

### Impaired Learning in Parkinsonians and the Effect of Levodopa Medication

Striatal dopamine deficiency in Parkinson's Disease is concerned with degeneration of dopaminergic neurons which results in smaller amounts of dopamine released. This affects both the baseline striatal dopamine concentration and phasic excursions of dopamine concentration that encode the reward prediction error. Our model predicts that lower dopamine concentration also leads to shortening of the pause in TAN activity, during which the phasic dopamine component drives reinforcement learning in the striatum. Using the model, we find that dopamine deficiency influences learning performance in the BG not only due to smaller magnitude of the learning signal, but also by decreasing the duration of the pause in TAN activity. From our simulation results, we found that 50% of dopamine deficiency in the model is sufficient to induce as poor learning performance as observed in Parkinsonians. This finding is consistent with the experimental data on striatal dopamine deficiency in Parkinson's Disease patients (Scherman et al., 1989) where it was reported that Parkinsonian symptoms appear when striatal dopamine deficiency exceeds 50%.

Levodopa is one of common treatments for early stage Parkinson's Disease patients (Brooks, 2008; Kalia and Lang, 2015). Levodopa administration increases Parkinson's Disease patient's UPDRS (Unified Parkinson's Disease Rating Scale) score by two or three times (Brooks, 2008; Beigi et al., 2016; Chen et al., 2016). In Gutierrez-Garralda et al.'s experiments (Gutierrez-Garralda et al., 2013), Parkinson's Disease patients were tested in the morning before taking their levodopa medicine to avoid levodopa effects on the results. According to a report, a standard dose of intravenous levodopa infusion increased the striatal dopamine level by 5–6 times (Zsigmond et al., 2014). Due to the lack of data, it is hard to know by how much the oral intake of levodopa increases dopamine concentration in the striatum. However, from the conventional dosage for Parkinson's Disease patients (Brooks, 2008), we can infer that oral levodopa may take more time to increase striatal dopamine levels and have less efficacy on striatal dopamine levels than intravenous levodopa infusion. In our simulations, levodopa 1.0 (2 times higher than baseline dopamine in 50% dopamine deficiency) caused the learning performance to recover close to the control levels (see Figure 5B). This effect is solely provided by the prolonged pause in TAN activity due to the levodopa-induced increase in baseline dopamine concentration. Interestingly, the extended pause duration at levodopa 1.0 is close to the one in control (no dopamine deficiency) conditions (see Figure 6C). The required increase of the baseline dopamine concentration by levodopa administration and the one predicted by the model is within a ballpark range.

### Alternative TAN Pause Mechanisms

In our model, the pause in TAN activity is induced by a cortico-thalamic excitatory input which causes after-hyperpolarization. However, other mechanisms for TAN pause generation have been proposed. For example, there exist inhibitory projections from GABAergic neurons in ventral tegmental area (VTA) to the cholinergic interneurons in nucleus accumbens (Brown et al., 2012). Brown et al. (2012) were able to generate a pause of TANs in nucleus accumbens by optogenetically activating VTA GABAergic projection neurons and link this to potentiation of associative learning.

Interestingly, regardless of how the pause is generated, our model would exhibit the same qualitative features of interactions between TAN activity and DA release. Indeed, TAN recovery from the pause would still depend on activation of depolarizing h-current negatively modulated by DA through D2 receptors. Therefore, TAN pause duration would positively correlate with DA concentration thus providing the same basis for our conclusions.

On a side note, GABAergic inhibition of TANs has not been found in dorsal striatum (Zhang and Cragg, 2017), which means that external inhibition cannot represents the primary mechanism of the pause in dorsal striatal TAN activity. The same lab has recently provided further evidence that the pause in TAN activity is associated with intrinsic properties of striatal cholinergic interneurons, induced by an excitatory input, mediated by potassium currents, and modulated by dopamine (Zhang et al., 2018).

## Materials and Methods

### The Model of TAN Activity

Our model describes the collective dynamics of a population of striatal tonically active neurons (TANs). The model represents the aggregate firing rate (activity) of the population treated as a smooth function of time *t* with TAN activity denoted by *V*_*TAN*_(*t*). The following differential equation governs its dynamics:

(1) $$\begin{array}{r} \tau_{TAN}\frac{dV_{TAN}\left(t\right)}{dt}+V_{TAN}\left(t\right)=\sigma \left(I_{TAN}\left(t\right)\right) \end{array}$$

where τ_*TAN*_ is a time constant, σ (*x*) = Θ (*x*)·tanh(*x*) is a sigmoid function, Θ (*x*) is Heaviside's function, and *I*_*TAN*_(*t*) is a term representing an aggregate input composed of intrinsic current inputs and synaptic inputs to the TAN population:

(2) $$\begin{array}{r} I_{TAN}\left(t\right)=W_{Thal}\cdot V_{Thal}\left(t\right)+Drv_{TAN}+I_{sAHP}\left(t\right)+I_{H}\left(t\right) \end{array}$$

Here *V*_*Thal*_(*t*) is a thalamic stimulus equal to 1 during stimulation and 0 otherwise, *W*_*Thal*_ is a synaptic weight of the thalamic input, *Drv*_*TAN*_ is a constant drive that defines the baseline firing rate, *I*_*sAHP*_(*t*) is a slow after-hyperpolarization current input, and *I*_*H*_(*t*) is an h-current input.

The slow after-hyperpolarization current *I*_*sAHP*_(*t*) is a hyperpolarizing current activated when the TAN activity exceeds certain threshold; the dynamics of this current are defined as

(3) $$\begin{array}{l} \tau_{sAHP}\frac{dI_{sAHP}\left(t\right)}{dt}+I_{sAHP}\left(t\right)=-g_{sAHP}\cdot \left(V_{TAN}\left(t\right)-\theta_{sAHP}\right)\\ \;\cdot \Theta \left(V_{TAN}\left(t\right)-\theta_{sAHP}\right) \end{array}$$

where τ_*sAHP*_ is a time constant, *g*_*sAHP*_ is the activation gain, and θ_*sAHP*_ is the threshold for activation.

In contrast to *I*_*sAHP*_, the depolarizing *h*-current *I*_*H*_(*t*) is activated when the TAN activity is below certain threshold, and its activation is modulated by the dopamine concentration. Its dynamics is defined by the following equation.

(4) $$\begin{array}{l} \tau_{H}\frac{dI_{H}\left(t\right)}{dt}+I_{H}\left(t\right)=-g_{H}\cdot \exp\left(-W_{DA}\cdot \left[DA\right]\left(t\right)\right)\\ \;\cdot \left(V_{TAN}\left(t\right)-\theta_{H}\right)\cdot \Theta \left(\theta_{H}-V_{TAN}\left(t\right)\right) \end{array}$$

where τ_*H*_ is a time constant, *g*_*H*_ is the activation gain, *W*_*DA*_ is the dopamine weight coefficient, [*DA*] is the concentration of striatal dopamine, and θ_*H*_ is the *h*-current activation threshold.

The temporal dynamics of striatal dopamine are defined by

(5) $$\begin{array}{l} \tau_{DA}\frac{d\left[DA\right]\left(t\right)}{dt}+\left[DA\right]\left(t\right)={\left[DA\right]}_{0}+RPE\cdot \left(1-\frac{V_{TAN}\left(t\right)}{\theta_{DA}}\right)\\ \;\cdot \Theta \left({\theta_{DA}-V}_{TAN}\left(t\right)\right) \end{array}$$

where τ_*DA*_ is the time constant, *RPE* is the reward prediction error, θ_*DA*_ is the nicotinic receptor threshold, [*DA*]_0_ is the baseline dopamine concentration.

To calibrate the model, we replicated experimental data published by Ding et al. (2010) who recorded TAN activity from sagittal slices of mice brains while stimulating either thalamic or cortical neurons while blocking D2 receptors with sulpiride or increasing dopamine levels by cocaine (Figure 3). All parameters were tuned to fit the experimental data and their values are listed below:

$$\begin{array}{l} \tau_{TAN}=20ms,W_{Thal}=4,Drv_{TAN}=0.3,\tau_{sAHP}=700ms,\\ g_{sAHP}=5,\theta_{sAHP}=0.3,\tau_{H}=700ms,\\ g_{H}=20,\theta_{H}=0.2,W_{DA}=1,\\ \tau_{DA}=20,\theta_{DA}=0.01,{\left[DA\right]}_{0}=1 \end{array}$$

To simulate the effect of sulpiride (Figure 3B) we set *W*_*DA*_ = 0 as sulpiride is a selective antagonist of dopamine D2 receptors. To simulate the effect of suppressed dopamine reuptake by cocaine (Figure 3C) we set [*DA*]_0_ to three times its control value [*DA*]_0_ = 3. We simulated blocking *h*-current (Figure 3D) by setting *g*_*H*_ = 0.

### Simulation of Behavioral Experiments

#### Integration of TAN-Dopamine Interactions Into the Model of Reward-Based Motor Adaptation

**Figure 7:**
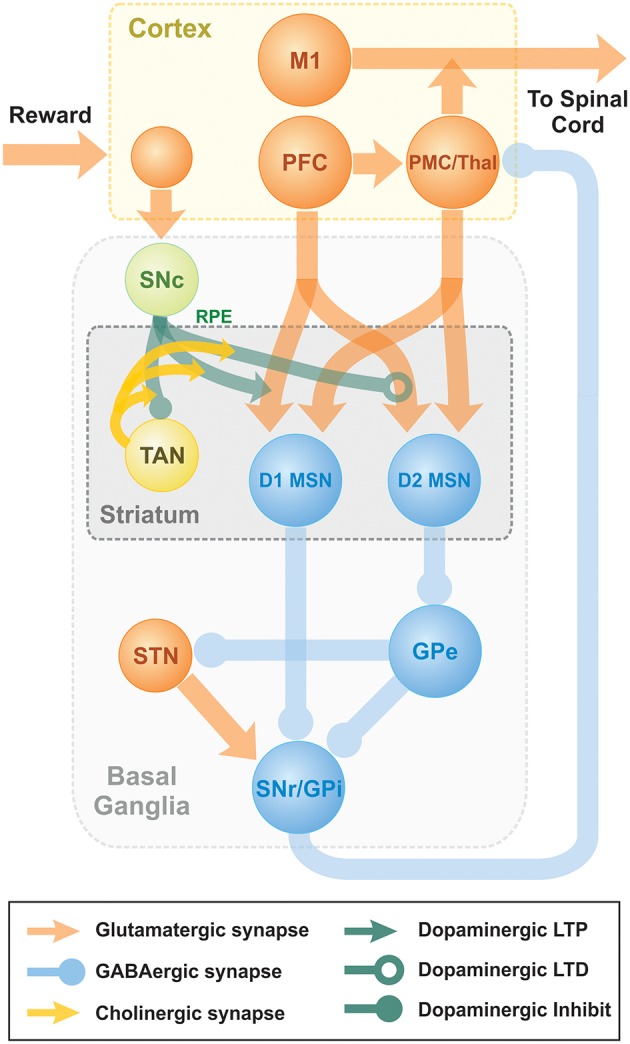
Schematic diagram of two-pathway of basal ganglia integrated with TAN model. Dopaminergic Substantia Nigra pars compacta signal represents the reward prediction error (reward prediction error). PFC, PreFrontal Cortex; M1, Primary Motor Cortex; PMC, PreMotor Cortex; MSN, Medium Spiny Neuron; SNr, Substantia Nigra pars Reticulata; GPi, 0Globus Pallidus internal; GPe, Globus Pallidus external; Substantia Nigra pars compacta, Substantia Nigra pars Compacta; STN, SubThalamic Nucleus.

Previously, we published a model able to reproduce key experiments concerned with non-error-based motor adaptation in the context of center-out reaching movements (Kim et al., 2017). The model included 3 modules: a 2 pathway (direct and indirect) BG module, a lower level spinal cord circuit module that integrated supra-spinal inputs with feedback from muscles, and a virtual biomechanical arm module executing 2D reaching movements in a horizontal plane (see Kim et al., 2017; Teka et al., 2017 for the details). The BG module was responsible for selection and reinforcement of the reaching movement based on reward provided. To study effects of TAN activities on dopaminergic signaling in the striatum, we integrated the model of TAN-dopamine interaction described above into the model of Kim et al. (2017). A schematic of the integrated model is shown in Figure 7.

The model of reinforcement learning in basal ganglia we used in this study was previously published and is described in details in Kim et al. (2017). Here, we only provide short qualitative description. Behavioral experiments studying reinforcement learning mechanisms assume that a choice must be made between several differentially rewarding behavioral options. Unlike decision-making tasks, motor learning does not imply a small or finite number of possible choices. The only constraint is the context of the task, e.g., reaching from a fixed initial position to an unknown destination. Our model has unlimited number of possible actions. As the context, we used center-out reaching movements performed in a horizontal plane. To calculate cortical activity corresponding to different movements, we explicitly solved an inverse problem based on the given arm kinematics. Accordingly, for every possible reaching movement we could calculate the corresponding motor program represented by the activity profiles of cortical inputs responsible for activation of different muscles. To describe different experiments, we define corresponding (arbitrarily large) sets of motor programs that define all possible behavioral choices (actions) in each experimental context.

The classical view of action selection is that different motor actions are gated by thalamocortical relay neurons. In the presented model, we assume that relay neurons can be activated at different firing rates, and their firing rates define contributions of different motor programs to the resulting motor response. More specifically, in our model cortical input to the spinal network is implemented as a linear combination of all possible motor programs in the given context with coefficients defined by the firing rates of corresponding thalamocortical relay neurons. This linear combination can be viewed as an aggregate input to the spinal network from the cortical motoneurons exhibiting activity profiles corresponding to different motor behaviors, e.g., reaching movements in different directions.

The classical concept of BG function is that the BG network performs behavioral choice that maximizes reward. This action selection process results in activation of thalamic relay neurons corresponding to the selected action and suppression of neurons gating other behaviors. Per this concept, each action is dedicated to specific neurons in different BG nuclei. Their focused interconnections form action-related loops which start at the cortex, bifurcate in the striatum into direct and indirect pathways converging on the internal Globus Pallidus (GPi), and feed back to the cortex through the thalamus. Action preference is facilitated by increased excitatory projections from sensory cortical neurons representing the stimulus to direct pathway striatal neurons (D1 MSNs). Suppression of unwanted competing actions is assumed to occur because of lateral inhibition among the loops at some level of the network in a winner-takes-all manner.

In the model, novel cue-action associations are formed based on reinforcement learning in the striatum. Eventually, the preferable behavior is reliably selected due to potentiated projections from the neurons in prefrontal cortex (PFC), activated by the provided stimulus, to D1 MSNs, corresponding to the preferred behavior. In technical terms, the output of basal ganglia model is the activation levels of thalamocortical relay neurons in response to the input from PFC neurons activated by visual cues. Each cure represents one of the possible reaching targets. These levels are used as coefficients of the linear combination of all possible actions which represents the motor program selected for execution. The resulting motor program is used to calculate the endpoint of the movement using neuro-mechanical arm model (Teka et al., 2017). Depending on the distance between the movement endpoint and the target position, the reward is calculated as dictated by the experimental context. This reward value is used to calculate the reward prediction error as a temporal difference between the current and previous reward values. The reward prediction error is used as the reinforcement signal (positive or negative deviation of dopamine concentration from its baseline levels) to potentiate or depress synaptic projections from PFC neurons, activated by the visual cue provided, to the striatal neurons, representing the selected actions. See details in Kim et al. (2017).

In Kim et al. (2017), the reinforcement learning is described as a trial-to-trial change in the synaptic weights of prefrontal cortico-striatal projections as follows:

(6) $$\begin{array}{r} \Delta W_{ji}^{1}=\lambda_{1}\cdot C_{j}\cdot D_{i}^{1}\cdot RPE-d_{w}\cdot W_{ji}^{1} \end{array}$$

(7) $$\begin{array}{r} \Delta W_{ji}^{2}=-\lambda_{2}\cdot C_{j}\cdot D_{i}^{2}\cdot RPE-d_{w}\cdot W_{ji}^{2} \end{array}$$

where: ${\Delta W}_{ji}^{1}$ and $\Delta W_{ji}^{2}$ are the changes in synaptic weights between PFC neuron *j* and D1- and D2-MSNs *i*, respectively, λ_1_ and λ_2_ are the learning rates, *RPE* is the reinforcement signal equal to the reward prediction error, *C*_*j*_ is the firing rate of PFC neuron *j*; $D_{i}^{1}$ and $D_{i}^{2}$ are the firing rate of D1- and D2- MSNs *i*, respectively, and *d*_*w*_ is a degradation rate.

In the integrated model, we assume that learning in the striatum is a continuous process defined by the deviation of dopamine concentration from its baseline value. Therefore, we replace the difference equations above with their differential analogs with reward prediction error replaced with the phasic component of the dopamine level:

(8) $$\begin{array}{r} \frac{d}{dt}W_{ji}^{1}={\bar{\lambda }}_{1}\cdot C_{j}\cdot D_{i}^{1}\cdot \left(\left[DA\right]\left(t\right)-{\left[DA\right]}_{0}\right)-{\bar{d}}_{w}\cdot W_{ji}^{1} \end{array}$$

(9) $$\begin{array}{r} \frac{d}{dt}W_{ji}^{2}=-{\bar{\lambda }}_{2}\cdot C_{j}\cdot D_{i}^{2}\cdot \left(\left[DA\right]\left(t\right)-{\left[DA\right]}_{0}\right)-{\bar{d}}_{w}\cdot W_{ji}^{2} \end{array}$$

Considering that dopamine concentration ([*DA*]) excurses from the baseline ([*DA*]_0_) during a short pause in TAN activity only, while the degradation process occurs continuously on a lot longer timescale, we can approximately rewrite these equations in a difference form by integrating over the pause duration:

(10) $$\begin{array}{r} \Delta W_{ji}^{1}=\;{\bar{\lambda }}_{1}\cdot C_{j}\cdot D_{i}^{1}\cdot \int \left(\left[DA\right]\left(t\right)-{\left[DA\right]}_{0}\right)dt-d_{w}\cdot W_{ji}^{1} \end{array}$$

(11) $$\begin{array}{r} \Delta W_{ji}^{2}=-{\bar{\lambda }}_{2}\cdot C_{j}\cdot D_{i}^{2}\cdot \int \left(\left[DA\right]\left(t\right)-{\left[DA\right]}_{0}\right)dt-d_{w}\cdot W_{ji}^{2} \end{array}$$

Where λ_1,2_ = λ_1,2_ · 0.00125 if [*DA*] ≥ [*DA*]_*o*_ or λ_1,2_ = λ_1,2_ · 0.0025 if [*DA*] < [*DA*]_*o*_.

All other parameters of BG model remain unchanged and can be found in Kim et al. (2017).

### Dopamine Deficiency Simulation

Striatal dopamine deficiency is caused by degeneration of dopamine producing neurons as observed in Parkinson's Disease patients. Parkinson's Disease is a long-term neurodegenerative disorder of the central nervous system that mainly affects the motor system. Shaking, rigidity, slowness of movements and difficulty with walking are the most obvious Parkinson's Disease symptoms so called parkinsonism or parkinsonian syndrome (Kalia and Lang, 2015). Motor learning is also impaired (Gutierrez-Garralda et al., 2013). Aging is also often accompanied by death of midbrain Substantia Nigra pars compacta neurons which causes parkinsonism-like motor disorders (Kalia and Lang, 2015).

Based on the above, we assume that dopamine deficiency results from a reduced number of dopamine neurons which produce proportionally smaller amount of dopamine. To simulate this condition, we multiply the right-hand side of the equation describing dopamine concentration dynamics

(12) $$\begin{array}{l} \tau_{DA}\frac{d\left[DA\right]\left(t\right)}{dt}+\left[DA\right]\left(t\right)=\alpha (RPE\cdot \left(1-\frac{V_{TAN}\left(t\right)}{\theta_{DA}}\right)\\ \;\cdot \Theta \left(\theta_{DA}-V_{TAN}\left(t\right)\right)+{\left[DA\right]}_{0}) \end{array}$$

by a coefficient α between 0 and 1 with α = 1 corresponding to 0% dopamine deficiency and α = 0 meaning 100% dopamine deficiency, i.e., no dopamine is produced at all. Fifty percent dopamine deficiency used in our simulations assumes that the coefficient used is α = 0.5, 30% deficiency corresponds to α = 0.7, etc.

### Levodopa Medication Simulation

Levodopa is an amino acid made by biosynthesis from the amino acid L-tyrosine (Knowles, 1986). Levodopa can cross the blood brain barrier whereas dopamine itself cannot and so it is naturally transferred into the brain via blood circulation (Wade and Katzman, 1975). Then levodopa as a precursor to dopamine is converted to dopamine by the enzyme called DOPA decarboxylase (aromatic L-amino acid decarboxylase) in the central nervous system (Hyland and Clayton, 1992). Thus, levodopa application increases overall dopamine concentrations in the brain. Levodopa medication is a clinical treatment for Parkinson's Disease patients as dopamine replacement to compensate for the dopamine deficiency. It is unclear whether levodopa improves the function of remaining dopamine neurons or affects baseline levels of dopamine in the brain only.

Our objective was to investigate if increasing the baseline dopamine concentration by levodopa without affecting the phasic dopamine release can improve learning performance in simulated Parkinson's Disease conditions. Thus, we mathematically describe the effect of levodopa medication by adding a constant term to the right-hand side of the equation for dopamine concentration

(13) $$\begin{array}{l} \tau_{DA}\frac{d\left[DA\right]\left(t\right)}{dt}+{\left[DA\right]}_{0}=\alpha (RPE\cdot \left(1-\frac{V_{TAN}\left(t\right)}{\theta_{DA}}\right)\\ \;\cdot \Theta \left(\theta_{DA}-V_{TAN}\left(t\right)\right)+{\left[DA\right]}_{0})+LDOPA \end{array}$$

where *LDOPA* is an increase in the baseline dopamine concentration due to levodopa administration. Correspondingly, to calculate the phasic component of dopamine dynamics in conditions of dopamine deficiency and/or levodopa medication for the baseline dopamine concentration, we use α[*DA*]_0_ + *LDOPA* instead of [*DA*]_0_.

## Simulation Environment

Our basic TAN activity-DA release interaction model was developed and simulated in Matlab. Then the model was implemented in C++ to integrate it into our previous model of reward-based motor adaptation described in detail in Kim et al. (2017). All simulations for behavioral experiments were performed using custom software in C++. The simulated data were processed in Matlab to produce figures. For behavioral experiments, we performed 75 simulations (25 before perturbation, 25 with perturbation, 25 after perturbation) per session and results of 8 sessions were averaged (see Kim et al., 2017 for more details).

## Author Contributions

TK, SM, YM, and IR: conceptualization; TK, SM, and YM: methodology; TK, RC, KH, WB, DT, SM, EL, and YM: validation; TK, RC, SM, and YM: formal analysis and software; TK, RC, KH, SM, and YM: investigation; SM, IR, and YM: resources; TK, RC, KH, WB, DT, EL, and SM: data curation; TK, RC, and YM: writing (original draft preparation); TK, RC, KH, WB, DT, EL, SM, IR, and YM: writing (review and editing); TK, RC, and SM: visualization; IR, and YM: supervision, project administration, and funding acquisition.

### Conflict of Interest Statement

The authors declare that the research was conducted in the absence of any commercial or financial relationships that could be construed as a potential conflict of interest.
